# Possible FDA-approved drugs to treat Ebola virus infection

**DOI:** 10.1186/s40249-015-0055-z

**Published:** 2015-05-04

**Authors:** Shu Yuan

**Affiliations:** College of Resources Science and Technology, Sichuan Agricultural University, Chengdu, 611130 China

**Keywords:** Ebola virus infection, Disseminated intravascular coagulation, Glycosylation inhibitors, Miglustat, Niemann-Pick C1 inhibitors, Toremifene

## Abstract

There is currently no effective treatment for the Ebola virus (EBOV) thus far. Most drugs and vaccines developed to date have not yet been approved for human trials. Two FDA-approved c-AbI1 tyrosine kinase inhibitors Gleevec and Tasigna block the release of viral particles; however, their clinical dosages are much lower than the dosages required for effective EBOV suppression. An α-1,2-glucosidase inhibitor Miglustat has been shown to inhibit EBOV particle assembly and secretion. Additionally, the estrogen receptor modulators Clomiphene and Toremifene prevent membrane fusion of EBOV and 50-90% of treated mice survived after Clomiphene/Toremifene treatments. However, the uptake efficiency of Clomiphene by oral administration is very low. Thus, I propose a hypothetical treatment protocol to treat Ebola virus infection with a cumulative use of both Miglustat and Toremifene to inhibit the virus effectively and synergistically. EBOV infection induces massive apoptosis of peripheral lymphocytes. Also, cytolysis of endothelial cells triggers disseminated intravascular coagulation (DIC) and subsequent multiple organ failures. Therefore, blood transfusions and active treatments with FDA-approved drugs to treat DIC are also recommended.

**Electronic supplementary material:**

The online version of this article (doi:10.1186/s40249-015-0055-z) contains supplementary material, which is available to authorized users.

## Multilingual abstracts

Please see Additional file 1 for translations of the abstract into the six official working languages of the United Nations.

## Review

**Figure 1:**
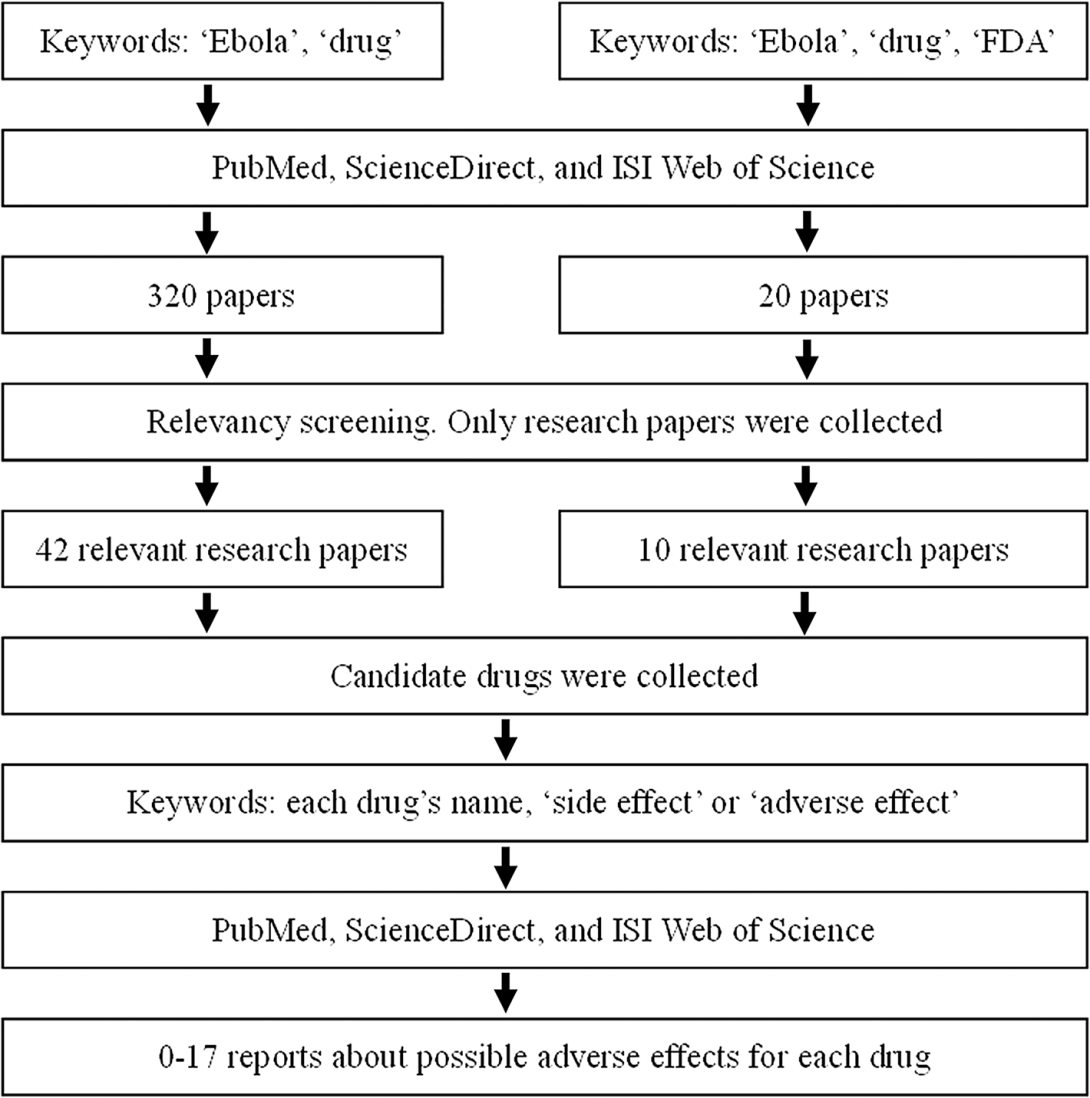
Flow chart algorithm for the literature search.

In the recent outbreak of the Ebola virus (EBOV) in Africa, more than 20,000 people were infected causing more than 8,000 deaths this year (recorded until January 14, 2015). No specific treatment for the Ebola virus is available, as of yet. Vaccines have been recently developed [1,2] and human trials are scheduled to begin shortly. However, there is still a long way before these vaccines can be applied clinically. Positively charged phosphorodiamidate morpholino oligomers (PMOplus) are effective in the treatment of EBOV [3]. BCX4430 is a type of adenosine analogue, which can also inhibit viral replication [4]. TKM-Ebola, the most promising gene therapy agent, has only recently entered phase I clinical trials [5]. Currently, neither antibodies nor existing drugs can directly relieve the hemorrhagic fever symptom, and they are usually ineffective when applied later than 4 days after infection (interferon-α at day 1 post-infection combined with an antibody mixture at the fourth day are also effective, but not any later) [6]. Furthermore, most of these are newly developed drugs (antibodies) without FDA approval and treatment may not be economically feasible for thousands of African patients (Figure 1 and Table 1).

**Table 1:** **Drugs for inhibiting EBOV replication**

**Drug generic name (trade name)**	**FDA approvement**	**Evidence in living animals with EBOV infections**	**Max human clinical dosage ≥ concentration to effective EBOV inhibition**	**Safety (side effects)**	**References**
**EBOV antibodies**	No (in phase I trial)	Yes	Not available	In assessing	[1,2,6]
**TIM-1 antibody**	No	No	Not available	Not available	[15,16]
**PMOplus**	No	Yes	Not available	Not available	[3]
**BCX4430**	No	Yes	Not available	Not available	[4]
**TKM-Ebola**	No (in phase I trial)	Yes	Not available	In assessing	[5]
**Ouabain**	No	No	Not available	Toxic in high levels	[14]
**Imatinib (Gleevec or Glivec)**	Yes	No	No	A little	[17]
**Nilotinib (Tasigna)**	Yes	No	No	A little	[17]
**Miglustat**	Yes	Yes	Yes (by oral admin.)	A little	[23]
**Benzylpiperazine adamantane diamide**	No	No	Not available	Not available	[26]
**Clomiphene (Androxal, Clomid or Omifin)**	Yes	Yes	Yes (by injection)	A little	[27,28]
**Toremifene (Fareston or Acapodene)**	Yes	Yes	Yes (by oral admin.)	A little	[27,28]
**Amiodarone (Cordarone, or Nexterone), Dronedarone (Multaq) or Verapamil (Calan or Isoptin)**	Yes	No	Not available	Risk of QT prolongation (cardiotoxicity)	[27,29]
**Amiloride (Midamor)**	Yes	No	Not available	A little	[30,31]
**Chloroquine (Aralen)**	Yes	No	Ineffective for primates	A little	[32,33]
**Favipiravir (Avigan)**	No	Yes	Suboptimal for primates	A little	[35]

Viral infection often leads to excessive host immune responses, which may cause death. In this case, the body attacks itself [7]. If the excessive immune response was restrained, the virus could possibly be cleared later by the body's immune mechanisms (if the virus is recognizable by the human immune system), similar to the influenza virus [7]. In the last year, I proposed an “Avian Influenza Cocktail Therapy” (AICT) to control excessive inflammation and inhibit viral replication [7]. Here I put forward a similar treatment protocol for Ebola virus infection, which includes two suggestions: (a) Combined inhibition of glycosylation of viral GP protein (by Miglustat, presumably) and EBOV intracellular receptor NPC1 (by Toremifene, presumably) which may limit viral spread and force the virus to be exposed under the monitoring of the host immune system; (b) Blood transfusion which may control EBOV-induced acute DIC, an excessive immune response.

## Methods

For the purpose of this scoping review, I conducted a literature search of peer reviewed papers in electronic databases for the period up to December 2014. The purpose of the search and literature review was to assemble published articles and reports associated with this review, as well as to identify any drugs to treat EBOV infection on any level (*in-vitro* cell culture, animal models or non-human primates) and their limitations. The three main databases used in the search procedure were PubMed, ScienceDirect, and ISI Web of Science. We employed the keywords: ‘Ebola’, ‘drug’, with or without ‘FDA’. These keywords were entered into the ‘Title’, ‘Abstract’ and ‘Keywords’ fields in the databases. Through this search, we obtained a total of 320 results without the keyword ‘FDA’ and 20 results with the keyword ‘FDA’. These were screened for relevancy, resulting in a total of 42 research papers without ‘FDA’ and 10 research papers with ‘FDA’ which were analyzed for this review (Figure 1). For each drug, its side-effects were further explored in the three main databases with the keyword ‘side effect’ or ‘adverse effect’ and the drug’s name (Figure 1).

## Results and discussion

### Current drugs and treatments

#### Antiserum transfer

Levels of neutralizing antibodies are always low in EBOV-infected patients, likely because of glycosylation of the viral surface glycoprotein GP [8,9]. On the other hand, GP glycosylation induces antibody-dependent viral enhancement (see next section for details) [10,11]. Therefore, simple transfer of antiserum from convalescing patients did not protect recipient patients. On the contrary, plasma or serum from convalescing patients undesirably enhanced the infection of primate kidney cells by the EBOV [10].

#### Interferon and drugs targeting VP24 protein

**Figure 2:**
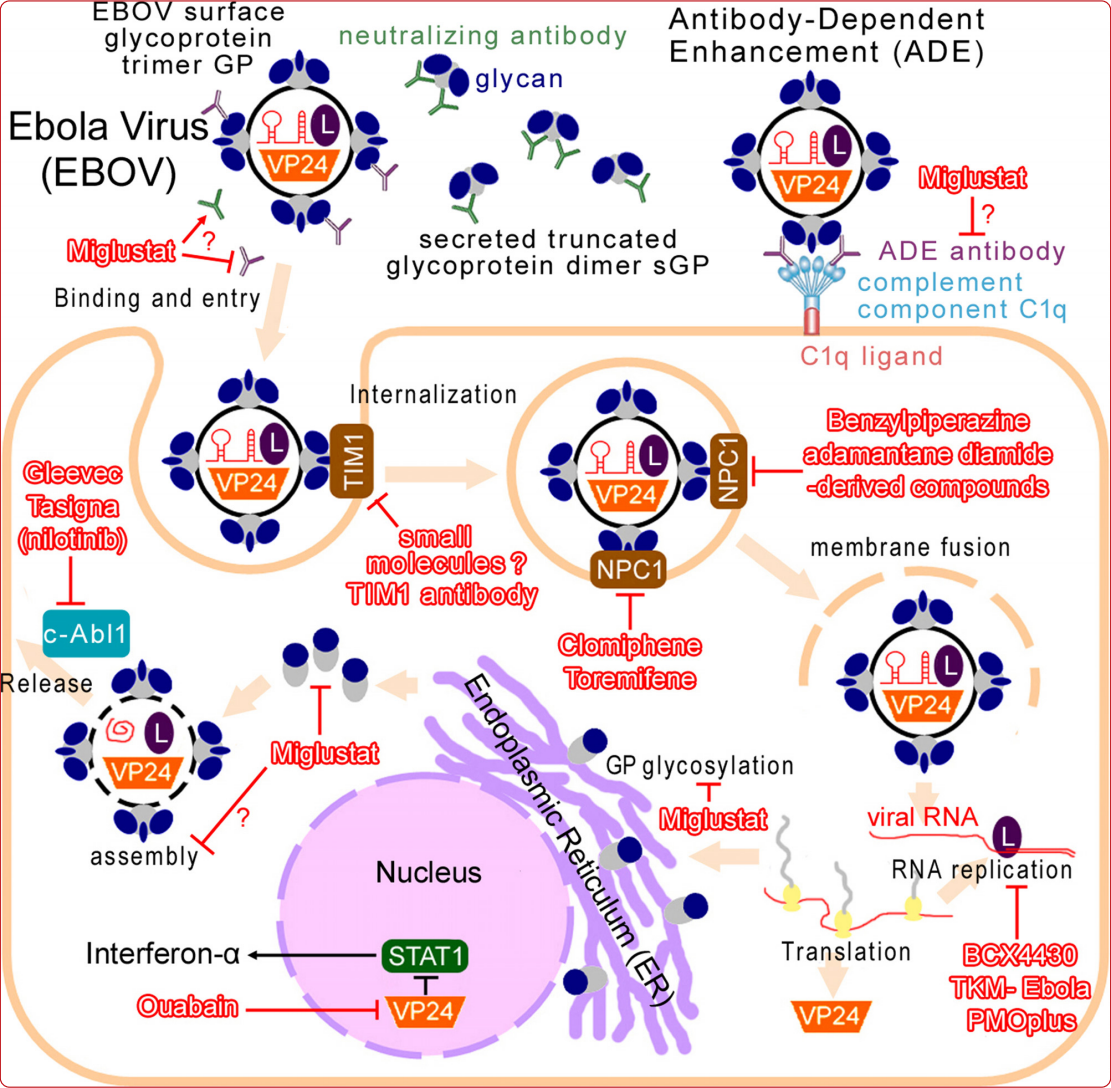
Model of the therapeutic mechanisms at the subcellular level: Drugs are shown with the stroke red color. EBOV, Ebola virus; L, viral RNA polymerase L protein. In addition to the viral surface glycoprotein (GP trimer), EBOV directs the production of large quantities of a truncated glycoprotein isoform (sGP dimer) that is secreted into the extracellular space. sGP can absorb anti-GP neutralizing antibodies (green ‘Y’) [9]. On the other hand, another antibody against glycosylated GP peptides is generated (purple ‘Y’), which enhances virus infection. The complement component C1q increases the likelihood of viral attachment to the cell surface [10,11]. Inhibition to GP glycans (dark-blue dot outside the GP protein) may reduce this antibody-dependent enhancement (ADE) ideally. Miglustat is a clinically-approved glycosidase inhibitor. Three derivates of Miglustat showed significant *in-vitro* antiviral activities against EBOV [23]. T-cell Ig and mucin domain 1 (TIM-1) and Niemann-Pick C1 (NPC1) are cellular receptors for EBOV [15,25]. The membrane fusion mediated by EBOV glycoproteins and viral escape from the vesicular compartment require the NPC1 protein [25]. Most NPC1 inhibitors are benzylpiperazine adamantane diamide derivates, non-FDA-approved drugs [26]. Recent studies showed that Clomiphene and Toremifene are novel NPC1 inhibitors and act as potential inhibitors of EBOV [27,28]. Viral VP24 protein inhibits nuclear import of the transcription factor STAT1, preventing interferon production [13]. Ouabain inhibits this process [14]. Two leukemia drugs Gleevec and Tasigna lower Ebola virus replication by inhibiting c-AbI1 tyrosine kinase, which is required for the release of Ebola virus particles [17].

The innate immune reaction after EBOV infection is characterized by a “cytokine storm,” with hypersecretion of numerous proinflammatory cytokines, chemokines, and growth factors, and by the noteworthy absence of antiviral interferon-α2 [12]. Viral VP24 protein binds karyopherin alpha nuclear transporters, inhibiting nuclear import of the transcription factor STAT1, therefore preventing interferon production [13]. However, a single treatment with interferon cannot cure EBOV infection, although interferon enhances the EBOV-specific adaptive immune response as well as inhibits viral replication [6]. Recently, researchers identified several proteins which interact with VP24 and found a small molecule inhibitor, Ouabain, which can inhibit EBOV replication in human lung cells [14]. However, Ouabain is not FDA-approved, and may be toxic in high concentrations. Besides, there is no experimental evidence for Ouabain in living animals infected with EBOV available so far (Figure 2 and Table 1).

#### Drugs targeting TIM-1

T-cell Ig and mucin domain 1 (TIM-1) protein is a cellular receptor for EBOV [15]. TIM-1 and related PS-binding proteins promote infection of diverse families of enveloped viruses [16]. Therefore, a monoclonal antibody against TIM-1 blocked EBOV binding and infection [15]. However, small molecules targeting TIM-1 have not yet been developed (Figure 2).

#### Drugs targeting c-AbI1

Two leukemia drugs, Gleevec (Imatinib) and Tasigna (Nilotinib) lower the Ebola virus’ replication ability by inhibiting c-AbI1 tyrosine kinase [17]. c-AbI1 is required for tyrosine phosphorylation of the Ebola matrix protein VP40, which is involved in the release of Ebola virus particles. Productive replication (TCID_50_) of the highly pathogenic Ebola virus Zaire strain was inhibited by 20 μM Nilotinib by up to four orders of magnitude [17]. However, the plasma concentrations of Imatinib or Nilotinib usually reached 2–3 μM at the normal dosages [18,19]. Even at the maximal dosages (two times of the normal dosage), the plasma concentrations (about 6 μM) are still far below the concentration for effective EBOV inhibition (20 μM) [17]. Besides, there is also no experimental evidence with living animals showing clinical validity to EBOV infection (Figure 2 and Table 1). Thus, neither Imatinib nor Nilotinib are currently used for EBOV therapy currently.

#### Glycosylation inhibitors

In addition to the viral surface glycoprotein (GP trimer), EBOV directs the production of large quantities of a truncated glycoprotein isoform (sGP dimer) that is secreted into the extracellular space. sGP can efficiently compete for anti-GP antibodies and therefore absorb anti-GP neutralizing antibodies [9]. The crystal structure of sGP showed that the glycan cap surrounding EBOV GP likely forms a shield that protects it from antigen-antibody binding, which is central to its immune evasion [8,20]. GP glycosylation inhibits neutralizing antibody production. Subsequently, another antibody against glycosylated GP peptides (the mucin-like domain) is generated, which enhances viral infection (infecting more endothelial cells and inducing extensive endothelial cell death). Complement component C1q enables binding between the virus-antibody complex and C1q ligands on the cell surface, promoting interaction between the virus and its receptor. Binding of the virus *via* the C1q molecule increases the likelihood of viral attachment to the cell surface [10,11]. Inhibition of GP glycans would ideally reduce this antibody-dependent enhancement (ADE).

The glycans that reside on the outside of GP are very complex in nature [8]. Therefore, glycosidases (especially the peptide-N-glycosidase) cannot reach the cleavage site efficiently. To target GP we must find another way to prevent (or change) the formation of the glycan cap. As of now, Miglustat (N-butyldeoxynojirimycin, American Actelion Pharms, for Gaucher’s Disease) is the only clinically-approved glycosidase inhibitor. It is a D-glucose analogue, which alters protein glycol processes within the endoplasmic reticulum (ER) and inhibits the interactions between folding glycoproteins and the ER chaperones calnexin and calreticulin by alpha 1,2-glucosidase inhibition [21,22]. The anti-viral effects of Miglustat have been well-documented in mammalian cells. It changes the viral envelope N-glycan composition and inhibits human immunodeficiency virus (HIV) entry (fusion) by a combined effect of a reduction in virion GP120 content and a qualitative defect of GP120 shedding and GP41 exposure [21,22]. For human hepatitis B virus (HBV), Miglustat prevents the secretion of enveloped DNA and causes the intracellular accumulation of excessive amounts of the envelope protein M [21].

EBOV GP proteins are heavily glycosylated and thus should be very sensitive to Miglustat. This assumption has been proved recently. Three derivates of Miglustat showed significant *in-vitro* antiviral activities against EBOV. Additionally, in a mouse model, high survival rates (50-70%) were observed for the Miglustat-derivate-treated animals [23]. Another recent study found that the removal of all GP1 N-glycans outside the mucin-like domain led to increases in protease sensitivity and antibody sensitivity, but also undesirably enhanced viral cell entry (fusion) [24]. Therefore, I presume that Miglustat may inhibit EBOV replication at the secretion or/and envelopment steps, like the mechanism to HBV. Besides, alteration to the EBOV GP glycan cap may also increase viral sensitivity to neutralizing antibodies, stimulate neutralizing antibody generation, and reduce the antibody-dependent enhancement (ADE, mentioned above), which would require further investigation (Figure 2 and Table 1).

#### NPC1 inhibitors

The endo/lysosomal cholesterol transporter protein Niemann-Pick C1 (NPC1) is the intracellular receptor to EBOV [25]. Cells defective for NPC1 function, including primary fibroblasts derived from human Niemann-Pick type C1 disease patients, are resistant to infection by Ebola virus. The membrane fusion mediated by EBOV glycoproteins and viral escape from the vesicular compartment require the NPC1 protein. Inhibition to NPC1 activity restricted the virus particles in cellular vesicular compartments [25]. However, most NPC1 inhibitors are benzylpiperazine adamantane diamide derivates; non-FDA-approved drugs [26]. *In vitro* screening of readily available approved drugs showed that selective estrogen receptor modulators Clomiphene and Toremifene are novel NPC1 inhibitors and act as potential inhibitors of EBOV (Figure 2 and Table 1) [27,28]. Although the survival rate of Toremifene treatment was only 50% (90% for Clomiphene), its effective concentration (2 μM) for EBOV inhibition (over 50%) was much lower than that of Clomiphene (10 μM) [28].

Besides Clomiphene and Toremifene, other cationic amphiphiles, including Amiodarone, Dronedarone, and Verapamil, also have been identified as potent inhibitors of the entry of the EBOV in a NPC1-dependent fashion [27,29]. However their effectiveness has only been proven in *in-vitro* cell culture assays. There is also no experimental evidence with living animals showing their clinical effectiveness against EBOV infections (Figure 2 and Table 1).

#### Other FDA-approved drugs

The Na^+^/K^+^ exchanger Amiloride inhibits virus uptake by macropinocytosis [30,31], however, experimental evidence with living animals is still lacking. Chloroquine induces alkalinization of endosomes and prevents the acid pH-dependent cleavage of Ebola virus GP by endosomal proteases cathepsin B and L [32,33]. However, proteolytic processing of the EBOV glycoprotein has been demonstrated to not be critical for EBOV replication in cell culture or nonhuman primates [34]. Favipiravir is a broad-spectrum inhibitor of viral RNA polymerase that is able to inhibit the replication of many RNA viruses. However, the survival benefit by oral administration was suboptimal in nonhuman primates. Only one of the six animals tested survived [35]. Antioxidants (such as N-acetylcysteine) could also be used to treat some viral infections, however clinical trials of some antioxidants in humans showed negative or ambiguous results or insignificant benefits [36]. These drug candidates for EBOV therapy have been recently summarized in a review [37].

### Drug effective concentration calculation for humans

#### Effective concentration calculation for Miglustat

25–75 mg/kg (50–150 μM/kg) Miglustat derivates at a 12-h interval were administrated for EBOV-infected mice and survival rates were achieved [23]. These dosages for mice are equal to 5.5-16.5 μM/kg (85–250 mg each time, 170–500 mg/day) for humans. The standard dosage for human Gaucher’s disease is 100 mg each, 3 times a day. And the maximum daily dose is 600 mg, which is a little higher than above maximum dosage (500 mg/day).

**Figure 3:**
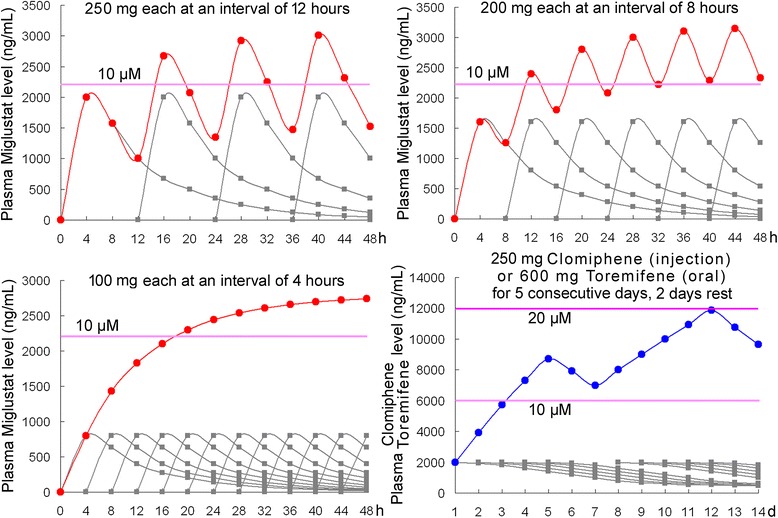
Accumulative plasma levels of Miglustat and Clomiphene (Toremifene) of different dosages and administration intervals in primates: For Miglustat, three conditions are calculated: 250 mg each at an interval of 12 hours (equal to the dosage and the interval for the mouse model); 200 mg each at an interval of 8 hours (the maximum dosage and the normal interval for humans); 100 mg each at an interval of 4 hours (as suggested here). Plasma level of 250 mg/day Clomiphene (injection) is approximately equal to that of 600 mg/day Toremifene (oral administration). Grey line indicates the supposed plasma level after single drug administration. Red line or blue line shows the calculated accumulative plasma levels of each drug. Purple line marks the concentration for effective EBOV inhibition as indicated in cell culture assay. h, hours. d, days.

Furthermore, we should not only calculate the dosage based on the body weight. Considering that endothelial cells are the main targets of EBOV, the drug plasma concentration may merit more relevance. 10 μM or higher concentration of Miglustat derivatives achieved inhibition ratios of over 50% to Ebola virus [23]. A single dose of 100 mg Miglustat administration resulted in the maximum plasma concentration of about 3–5 μM (influenced by food-intake). The plasma concentration reaches a maximal value within 4 hours, while the half-life time t_1/2_ is approximately 8 hours [38]. If 100 mg Miglustat is administered every 4 hours, 6 times a day, the plasma concentration would be stabilized at approximately 10 μM after 24 hours (contrastingly 12-hour interval causes a large fluctuation; Figure 3). Sustained high levels of Miglustat would inhibit viral replication persistently, and its curative effect would be much better than the situation with a large fluctuation, where the virus can be replicated intermittently. The side-effects of Miglustat include tremors, diarrhea, numbness, thrombocytopenia, and some gastrointestinal reactions [38]. This low-dose, short-interval drug-administration method may help to reduce these adverse reactions. If 200 mg Miglustat is administered every 8 hours, 3 times a day, the plasma concentration will be also higher than 10 μM after 24 hours (Figure 3), however this dose could lead to increased adverse reactions.

#### Effective concentration calculation to Clomiphene and Toremifene

A high level of 60 mg/kg (100 μM/kg, 2-day interval) Clomiphene/Toremifene was used for EBOV-infected mice and high survival rates were achieved [28]. This dosage for mice is equal to 11 μM/kg (400 mg/day, 2-day interval) for humans.

A single dose of 250 mg Clomiphene (the maximum daily dose) administration resulted in a maximum plasma concentration of about 3–4 μM. The minimum concentration required for effective EBOV inhibition is 10 μM [28]. However, Clomiphene is degradated very slowly (t_1/2_ is 5–7 days) [39], and therefore, a plasma concentration of 10 μM would be achieved after a second administration of the drug (Figure 3).

This plasma concentration of Clomiphene can only be achieved via intravenous injection. For Clomiphene, the plasma concentration by oral administration is about 27–50 times lower than the plasma concentration by intravenous injection [40]. In a mouse model, Clomiphene was also applied by the intraperitoneal injection method [28]. Considering that Clomiphene injection is not frequently used and inconvenient for African patients, oral administration of Toremifene could be adopted instead. A plasma concentration of 600 mg Toremifene by oral administration is approximately equal to the plasma concentration of 250 mg Clomiphene by intravenous injection (14 μM on days 4; Table 1 and Figure 3) [41]. The side-effects of Toremifene include hidrosis, metrorrhagia, pruritus, fatigue, dizziness, headache, depression, and other neurological symptoms, which are less severe than Clomiphene [39].

### Suggestion of Miglustat and Toremifene combination

Here I suggest the combination of both Miglustat and Toremifene to treat and possibly cure EBOV infection. Miglustat works at the secretion/envelopment step [23], while Toremifene functions at the entry/fusion step [28]. They act through different mechanisms and thus should be used in combination. A single use of Miglustat cannot inhibit virus replication significantly (50-70% survival rates were observed for the Miglustat-derivate-treated animals, but not for Miglustat itself) [23]. While application of Toremifene alone could neither alter GP glycosylation nor excite neutralizing antibody generation, and therefore the remnant virus cannot be cleared efficiently. Only through this combination, the lethal EBOV infection may be cured. Toremifene is a NPC1 inhibitor [27,28]. High levels of NPC1 inhibitors may cause a symptom similar to Niemann-Pick disease, due to NPC1-inhibition-induced cholesterol and sphingomyelin accumulation [42]. Luckily, Miglustat is supposed to alleviate Niemann-Pick neurological symptoms by inhibiting sphingomyelin synthesis [43]. In other words, Miglustat may partly alleviate the side-effects of Toremifene.

### Disseminated intravascular coagulation and possible treatment options

#### Disseminated intravascular coagulation (DIC)

Although endothelial cells are the targets of EBOV, the vascular endothelium remains relatively intact even at terminal stages of disease [44]. The hemorrhagic fever is not the direct result of EBOV-induced cytolysis of endothelial cells, and is likely triggered by some immune-mediated mechanisms: The virus itself and its toxins damage vascular endothelial cells, induce activator XII, kallikrein, and bradykinin, further activating the coagulation system [45]. Bradykinin also causes blood vessels to dilate (vasodilation) and therefore may cause hypotension and shock [46]. DIC is subsequently induced [47,48].

Prolonged prothrombin and partial thromboplastin [45] suggest microcirculation disturbance and the existence of these micro-thrombi during EBOV-infection. EBOV-infection also induces a dramatic rise in circulating D-dimers [45], indicating hyperfibrinolysis. Thus, both anticoagulants (heparin or protein C) and anti-fibrinolytic drugs (tranexamic acid) should be used for DIC patients to prevent later multiple organ failure [47,48].

#### Blood transfusion

Acute DIC develops into the consumptive and hypocoagulable stage very quickly, accompanied by acidosis [47]. Mechanical injuries to red blood cells and hemolysis subsequently cause hemoglobin release, while free heme causes severe oxidative damage [47]. DIC patient need fresh blood to supply erythrocytes, fibrinogen, and coagulation factors (including platelets). Moreover, blood transfusion also helps to balance serum electrolytes and reduce oxidative stress. The innate immune reactions after EBOV infection have been characterized as a “cytokine storm”. Great immunosuppression occurs later, which is characterized by very low levels of circulating cytokines produced by T lymphocytes and by massive apoptosis of peripheral CD4 and CD8 lymphocytes [12]. Blood transfusion could supply these cytokines and T lymphocytes and therefore enhance the patient’s immune system.

#### Haptoglobin

Adverse clinical effects associated with excessive free hemoglobin can be attributed to several specific structural and biochemical properties of the hemoglobin molecule, and are caused by the following four mutually interacting mechanisms: (a) extravascular translocation of hemoglobin, which is a principal requirement for hemoglobin and hemin to be able to induce their adverse reactions in tissues; (b) nitric oxide and oxidative reactions; (c) release of free hemin; and (d) molecular-signaling effects of hemin. Haptoglobin can neutralize hemoglobin and hemin and scavenge nitric oxide and physiologic oxidants, preventing hemolytic transfusion reactions and hemolysis-induced acute renal failure [49,50]. A human plasma-derived haptoglobin product has been approved for clinical use in Japan since 1985. The effective haptoglobin doses ranges from 3 g to > 20 g [50]. Haptoglobin may be very helpful for the late-stage patients.

## Conclusions and perspective clinical trials

Among the possible FDA-approved drugs to treat Ebola virus infection, Miglustat, Clomiphene, and Toremifene are the most promising, with preclinical evidence in living animals. Three derivates of Miglustat showed significant in vitro antiviral activities against EBOV. In a mouse model, significant survival rates (50-70%) were observed for their treatments. The survival rate of mice was 50-90% after Clomiphene/Toremifene treatments. However, the uptake efficiency of Clomiphene by oral administration is very low. Thus, oral administration of Toremifene is recommended. Miglustat works at the secretion/envelopment step, while Toremifene functions at the entry/fusion step. They act through different mechanisms and thus should be used in combination. A Single dose of Miglustat cannot inhibit virus replication significantly. Meanwhile, application of Toremifene alone cannot initiate neutralizing antibody generation, and therefore the remnant virus cannot be cleared efficiently. Only through this combination, the lethal EBOV infection may be cured.

The effective dosages of Miglustat and Toremifene for mice were much higher than those clinically used for humans. Here I put forward a hypothetical treatment protocol with cumulative uses of both Miglustat and Toremifene to control EBOV effectively and synergistically. A comprehensive treatment protocol to EBOV infection is proposed (Table 2). Miglustat and Toremifene are FDA-approved drugs with oral availability, good safety, and tolerability profiles and a long history of use. The oral availability of these drugs offers great utility in the resource-constrained geographical regions where outbreaks of EBOV infection occur. With the evidences in animal experiments and the effective plasma concentration calculated here, this treatment protocol may be ready for human clinical trials. However, given the constraints of the FDA animal rules and the World Health Organization (WHO) guidance, studies in non-human primates to assess this treatment strategy are usually required before the advancement to human clinical trials. There needs to be some level of efficacy demonstrated in non-human primates through an administration route and dosages similar to those that would be used in humans. Furthermore, with all combinational therapies, there is a potential antagonistic interaction(s) and/or potential increase in cytotoxicity or toxicity in the animal model, which should be also clarified before entering human clinical trials. Miglustat is a D-glucose analogue, while Toremifene is a cationic amphiphile (a dimethylethanamine derivate with three benzene rings). Therefore, there should be no direct interaction between the two drugs. Nevertheless, considering the severity of the current outbreak, I hope the treatment protocol could be approved by the World Health Organization (WHO) and applied to patients tentatively (phase I clinical trial). Alternatively, tests in non-human primates should be carried out immediately before the human trial.

**Table 2:** **Suggested treatment protocol to EBOV infection**

**Stage**	**Symptoms**	**Suggested drugs/treatments**	**Matters needing attention**
I. 2–3 days after onset	Nausea and vomiting, diarrhea and mucobloody stool, long-lasting diarrhea [46]	a) Miglustat (100 mg each at an interval of 4 hours)	a,b) Until fully viral clearance. c) If haemorrhage occurs, see Stage II.
		b) Toremifene (600 mg/day for 5 consecutive days, 2 days rest)	
		c) Water and electrolyte supply	
II. 4–5 days after onset	Hematemesis and melena, injection area bleeding, hemorrhinia, hemoptysis, sustained fever, accompanying myocarditis or pneumonia [46]	a) Miglustat (6 × 100 mg/day)	a,b) Until fully viral clearance. c) One or two more blood transfusions in the later days, if symptoms persist. If acute DIC occurs, see Stage III.
		b) Toremifene (600 mg, 5 days)	
		c) 200–400 ml blood transfusion	
III. 6–7 days after onset	Measles-like maculopapular rash at shoulders, palms and feet, then spreading throughout the body, desquamation several days later [46]	a) Miglustat (6 × 100 mg/day)	a,b) Until fully viral clearance. c) One or two more blood transfusions. DIC must be treated to prevent multiple organ failures.
		b) Toremifene (600 mg, 5 days)	
		c) 400–800 ml blood transfusion (heparin and tranexamic acid may be used)	
IV. 8–9 days after onset	Possible kidney failure or liver failure, orchitis, orchiatrophy, et al. [46]	a) Miglustat (6 × 100 mg/day)	a,b) Until fully viral clearance. c) One or two more blood transfusions. Massive blood transfusion or hemodialysis may be adopted if available.
		b) Toremifene (600 mg, 5 days)	
		c) 800 ml or more blood transfusion (heparin and tranexamic acid should be used)	
		d) 6–20 g human plasma haptoglobin (if available) [51]	

Notes: (1) Miglustat and Toremifene should be used for patients in the latent period upon diagnosis of EBOV infection. (2) Miglustat may not be replaced by Miglitol or other analogues without side chain alkylation [23]. (3) If 100 mg Miglustat at an interval of 4 hours is not feasible, 200 mg Miglustat at an interval of 8 hours may be applied instead (as calculated in Figure 2). (4) Ebola infections progress very fast, thus the virus replication should be inhibited in the first time. The low-dose, short-interval drug-administration method should not be applied for Toremifene (as calculated in Figure 2). (5) Blood transfusion is not obligatory, because it may be not feasible on the large scale. Blood transfusion Tranexamic acid may be replaced by 4-aminomethyl benzoic acid or 6-amino acetic acid. Adequate heparin must be used before the application of anti-fibrinolytic drugs [47,48]. (6) Reduced dosages should be adopted for children according to their body weight.

Because of its high mortality rate and infectious nature, EBOV usually causes psychological panic among the patients and the public that is more serious than the disease itself. In Africa, some EBOV patients refuse treatment and some other patients are hidden by their families, because of psychological panic [51]. However, these actions have accelerated viral spread and made epidemic prevention very difficult. Thus, if Miglustat and Toremifene, two FDA-approved drugs, are effective for humans, the panic may be eliminated and the current outbreak may be better controlled, in addition to potentially saving thousands of lives.

## Additional file

Additional file 1:
**Multilingual abstracts in the six official working languages of the United Nations.**

## Abbreviations

ADE: Antibody-dependent enhancement
AICT: Avian influenza cocktail therapy
DIC: Disseminated intravascular coagulation
EBOV: Ebola virus
ER: Endoplasmic reticulum
FDA: Food and drug administration
HBV: Human hepatitis B virus
HIV: Human immunodeficiency virus
NPC1: Niemann-Pick C1
PMOplus: Positively charged phosphorodiamidate morpholino oligomers
TCID_50_: Tissue culture infective dose 50%
TIM-1: T-cell Ig and mucin domain 1
WHO: World Health Organization
