# Supplementary material for: Possible FDA-approved drugs to treat Ebola virus infection
Source: Infect Dis Poverty. 2015 May 4;4:23. doi: 10.1186/s40249-015-0055-z (PMC4432825; doi:10.1186/s40249-015-0055-z)
Supplement: Additional file 1: — Multilingual abstracts in the six official working languages of the United Nations. [file 40249_2015_55_MOESM1_ESM.pdf]

Translation of the abstract into the six official working languages of the United Nations

أدوية قد تحصل على موافقة هيئة الأغذية والأدوية الأمريكية لعلاج العدوى بفيروس الايبولا

شو يوان

الملخص

لا يوجد في الوقت الحالي علاج فعال لفيروس الإيبولا. معظم الأدوية واللقاحات التي تم تطويرها حتى اليوم لم تحصل على الموافقة للتجارب على البشر. هناك عقاران حاصلان على موافقة هيئة الأغذية والأدوية الأمريكية (FDA) وهما مثبطا تيروزين كيناز c-AbI1 المعروفين باسم Gleevec وTasigna يمكنهما منع إطلاق الأجزاء الفيروسية، ولكن جرعتهما السريرية أقل بكثير من الجرعة المطلوبة لكبت فيروس الايبولا بطريقة فعالة. أظهر مثبط ألفا 1، 2 غلوكوزيداز المعروف باسم Miglustat قدرة على تثبيط تجمع أجزاء فيروس الايبولا وإفرازها. إضافة إلى ذلك، تمنع معدِّلات مستقبل الاستروجين كلوميفين Clomiphene وتوريميفين Toremifene التحام غشاء فيروس الايبولا وقد تمكن 50 – 90% من الفئران المعالجة من البقاء على قيد الحياة بعد العلاج بواسطة كلوميفين/توريميفين. ولكن تبقى فعالية قبط الكلوميفين بعد إعطائه عن طريق الفم منخفضة جدا. لذا، أقترح بروتوكولا علاجيا نظريا لعلاج العدوى بفيروس الايبولا بالاستعمال التراكمي لكل من Miglustat و Toremifene لتثبيط الفيروس بفعالية وتآزرية. تحفّز العدوى بفيروس الايبولا الموت الخلوي المبرمج الجسيم في اللمفاويات المحيطية. إضافة إلى ذلك، يستهدف الانحلال الخلوي للخلايا البطانية التخثر المنتشر داخل الأوعية (DIC) وما يليه من فشل الأعضاء المتعدد. لذا، يوصى أيضا بنقل الدم وعلاج التخثر المنتشر داخل الأوعية الفعال بالأدوية الحاصلة على موافقة هيئة الأغذية والأدوية الأمريكية.

Translated from English version into Arabic by Lina SM, through

TRANSLATORS
WITHOUT BORDERS

可能的治疗埃博拉病毒感染的 FDA 获批药物

袁澍

摘要

当前真对埃博拉病毒感染还没有特效药。现有的药物或疫苗都只是在研发中，未被批准直接用于人体临床。格列卫和尼洛替尼是两种美国食品药品监督局（FDA）批准的 c-AbI1 络氨酸激酶抑制剂，它们可以抑制病毒颗粒的释放。但是，它们的临床用药规定剂量达不到有效抑制埃博拉病毒复制的要求。α-1,2-葡萄糖苷酶抑制剂米格鲁特可以抑制病毒的装配和分泌。此外，雌激素受体调节剂克罗米芬和托瑞米芬可以阻止病毒与宿主细胞的膜融合，在小鼠中这两种药可以显著提高小鼠感染埃博拉病毒后的成活率到 50–90%。但是克罗米芬的口服吸收效率非常低。因此，我在这里提出一个治疗假说：米格鲁特和托瑞米芬联用，并且采取积累用药法以有效抑制病毒。埃博拉病毒感染造成患者外周淋巴细胞大量死亡。与此同时，血管内皮细胞的损伤还诱导弥散性血管内凝血（DIC）和后期严重的多器官衰竭。因此，适当的输血疗法和针对 DIC 开展积极的药物治疗也是值得推荐的。

Translated from English version into Chinese by Yuan Shu

Médicaments autorisés par la FDA potentiellement utilisable pour traiter l’infection par le virus Ébola

Shu Yuan

Résumé

Il n’existe pas actuellement de traitement efficace contre le virus Ébola. La plupart des médicaments et vaccins développés à ce jour n’ont pas encore franchi le cap de l’autorisation pour les essais chez l’homme. Deux inhibiteurs de la tyrosine-kinase c-Abl approuvés par la FDA (Food and Drug Administration) américaine, Glivec et Tasigna, bloquent la libération de particules virales. Leur posologie clinique est toutefois très inférieure à la dose nécessaire pour inhiber efficacement le virus Ébola. Le miglustat, un inhibiteur de l’α-1,2-glucosidase, a une action inhibitrice démontrée sur l’assemblage et de la sécrétion de particules de virus Ébola. En outre, deux modulateurs des récepteurs aux œstrogènes, le clomifène et le torémifène, empêchent la fusion du virus à la membrane ; le taux de survie de souris traitées avec ces molécules est compris entre 50 % et 90 %. L’efficacité d’absorption du clomifène administré par voie orale est cependant très faible. L’auteur propose donc un protocole de traitement hypothétique pour traiter l’infection par le virus Ébola en combinant miglustat et torémifène afin d’obtenir une synergie permettant d’inhiber efficacement le virus. L’infection par le virus Ébola provoque une apoptose massive des lymphocytes périphériques. Par ailleurs, la cytolyse des cellules endothéliales déclenche une coagulation intravasculaire disséminée (CID) et, de ce fait, la défaillance de nombreux organes. On peut donc aussi recommander des transfusions sanguines et un traitement actif contre la CID avec des médicaments autorisés par la FDA.

Translated from English version into French by Suzanne Assenat, through

TRANSLATORS
WITHOUT BORDERS

Возможные лекарства, одобренные Управлением по контролю за качеством пищевых продуктов и лекарственных препаратов, для лечения инфекций, вызванных вирусом Эбола

Шу Юань

Реферат

На сегодняшний день еще не найдено эффективное средство против вируса Эбола. Большинство лекарственных средств и вакцин, разработанных к настоящему времени, еще не получили утверждения для проведения клинических исследований с участием людей. Два ингибитора c-AbI1 тирозинкиназы, одобренных Управлением по контролю за качеством пищевых продуктов и лекарственных препаратов, гливек (Gleevec) и Tasigna блокируют выпуск вирусных частиц, однако, их клинические дозы намного ниже доз, требующихся для эффективного подавления вируса Эбола. Было показано, что ингибитор α-1,2 глюкозидазы миглустат (Miglustat) задерживает сборку и выделение частиц вируса Эбола. Кроме того, модуляторы рецепторов эстрогена кломифен (Clomiphene) и торемифен (Toremifene) предотвращают мембранный синтез вируса Эбола, и 50–90% подвергнутых лечению кломифеном/торемифеном мышей выжили. Однако, при оральном введении кломифена эффективность его поглощения очень низкая. Следовательно, я предлагаю возможный протокол лечения инфекций, вызванных вирусом Эбола, включающий использование миглустата и торемифена для эффективного и взаимоусиливающего

подавления вируса. Вызванные вирусом Эбола инфекции порождают массивный апоптоз периферических лимфоцитов. В дополнение, цитолиз эндотелиоцитов приводит к возникновению синдрома диссеминированной внутрисосудистой коагуляции (DIC) и к последующей функциональной недостаточности многих органов. Следовательно, рекомендуются также переливания крови и активное лечение лекарствами, одобренными Управлением по контролю за качеством пищевых продуктов и лекарственных препаратов.

Translated from English version into Russian by Alena Hrybouskaya, through

TRANSLATORS
WITHOUT BORDERS

Posibles medicamentos aprobados por la FDA para el tratamiento de la infección por el virus del ébola

Shu Yuan

Resumen
En estos momentos no existe un tratamiento efectivo para el virus del ébola (EVE). La mayoría de los medicamentos y vacunas desarrollados hasta la fecha no han sido aprobados para su ensayo en seres humanos. Dos medicamentos aprobados por la Administración de Alimentos y Medicamentos (FDA), Gleevec y Tasigna, inhibidores de la tirosina quinasa c-AbI1, bloquean la liberación de partículas virales. Sin embargo, las dosis clínicas son mucho más bajas que las dosis requeridas para una supresión efectiva del EVE. Un inhibidor de la alfa-1,2 glucosidasa, Miglustat, ha mostrado la capacidad de inhibir el esamblaje y secreción del EVE. Además, los moduladores de estrógeno clomifeno y toremifeno previenen la fusión de la membrana del EVE y 50 a 90% de los ratones tratados sobrevivieron luego del tratamiento con clomifeno/toremifeno. Si embargo, la eficiencia de absorción del clomifeno cuando seadministra por vía oral es muy baja. Por lo tanto, propongo un protocolo hipotético para el tratamiento del EVE con el uso acumulativo del miglustat y el toremifeno en conjunto para inhibir el virus de manera efectiva y sinérgica. La infección por el virus del ébola induce una apoptosis masiva de linfocitos periféricos. Asimismo, la citólisis de células endoteliales desencadena coagulación intravascular diseminada (CID) y la consiguiente insuficiencia de múltiples órganos. Por lo tanto, también se recomiendan las transfusiones de sangre y los tratamientos activos con medicametos aprobados por la FDA para tratar la CID .

Translated from English version into Spanish by Maria Alejandra Aguada, through

TRANSLATORS
WITHOUT BORDERS
